# Supplementary material for: Rapid Degradation of Chlortetracycline Using Hydrodynamic Cavitation with Hydrogen Peroxide
Source: Int J Environ Res Public Health. 2022 Mar 31;19(7):4167. doi: 10.3390/ijerph19074167 (PMC8998951; doi:10.3390/ijerph19074167)
Supplement: Supplementary file 1 [file ijerph-19-04167-s001.zip › ijerph-1614731-supplementary.pdf]

SUPPORTING INFORMATION

Rapid Degradation of Chlortetracycline Using Hydrodynamic Cavitation with Hydrogen Peroxide

Chen Meng[a], Min Meng[a], Xun Sun[b], Congcong Gu[a], Huiyun Zou[a], Xuewen Li[a*]

[1] Department of environment and health, School of Public Health, Cheeloo College of Medicine, Shandong University, Jinan, Shandong, 250012, China
[2] Key Laboratory of High Efficiency and Clean Mechanical Manufacture, Ministry of Education, School of Mechanical Engineering, Shandong University, Jinan, Shandong, 250061, China
* Correspondence: E-mail address: lxw@sdu.edu.cn. Tel: +86-0531-88382138

Text S1

1. Extent of Degradation of CTC related to Fenton

1.1. Preparation of experiments

Hydrogen peroxide(H_2O_2), Fenton and hydrodynamic cavitation (HC) combined with Fenton processes were used to treat CTC solution of 80mg/L. Referring to previous study, the ratio of [Fe^{2+}] / [H_2O_2] in Fenton and HC combined with Fenton processes was set as 1/2 and the concentration of H_2O_2 of experiments was 4mM. The pH of Fenton and HC combined with Fenton process was 2.0-3.0 and the pH of H_2O_2 process was 6.0. The extent of degradation of CTC for different treatments was performed for up to 30 min of reaction time and samples were removed to analyze for the extent of degradation at 5, 10, 20 and 30 min in each process.

1.2. Extent of Degradation of CTC

The extent of degradation rates for using H_2O_2, Fenton alone, and HC combined with Fenton were depicted in Figure S1. It can be observed the combined process has

a higher degradation efficiency compared with the treatments of using H_2O_2 and Fenton alone. The degradation rate of CTC was 88.2% and 88.5% at 5min and 30min, respectively, which is similar to that of HC combined with H_2O_2.

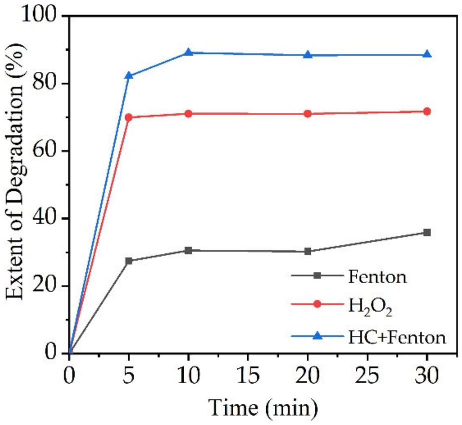

Figure S1. Extent of degradation of CTC by H_2O_2, Fenton, and HC combined with Fenton within 30 min.
